# Acute-on-Chronic Liver Failure: Causes, Clinical Parameters, and Predictors of Mortality

**DOI:** 10.7759/cureus.52690

**Published:** 2024-01-21

**Authors:** Fatima Hafsa, Zao Iman Chaudary, Owais Tariq, Zainab Riaz, Aamir Shehzad, Muhammad Irfan Jamil, Iqra Naeem

**Affiliations:** 1 Gastroenterology, Lahore General Hospital, Lahore, Lahore, PAK; 2 Internal Medicine, Doctors Hospital Lahore, Lahore, PAK; 3 Medicine, Milvik Bima Pakistan, Lahore, PAK; 4 General Medicine, Lahore General Hospital, Lahore, PAK; 5 Gastroenterology, Lahore General Hospital, Lahore, PAK; 6 Nephrology, Lahore General Hospital, Lahore, PAK; 7 Psychiatry and Behavioral Sciences, Lahore General Hospital, Lahore, PAK

**Keywords:** clif-sofa score, model for end stage liver disease (meld), chronic liver disease (cld), survival rate, mortality, acute-on-chronic liver failure (aclf)

## Abstract

Objectives

This study aimed to identify the causes, clinical characteristics, and 28-day in-hospital mortality predictors in patients with acute-on-chronic liver failure (ACLF).

Methods

A cross-sectional study enrolled sixty-four patients aged 18-70 years with acute-on-chronic liver failure. The study was conducted at the Gastroenterology Department, Lahore General Hospital. The study classified ACLF according to the criteria of the European Association for the Study of the Liver - Chronic Liver Failure (EASL-CLIF). Patients were followed for 28 days for mortality outcomes. The outcomes between Survivor and Non-survivor groups were compared using the Chi-Square/Fisher's Exact Test for categorical variables and the Mann-Whitney U test for continuous variables.

Results

In this study, age and duration of chronic liver disease were not significantly different between survivors and non-survivors. The etiology of liver disease and ACLF causes had no impact on 28-day mortality. Non-survivors had lower mean arterial pressure, and higher mortality was linked with lower Glasgow Coma Scale scores, upper gastrointestinal bleeding, and Grade IV hepatic encephalopathy. Significant differences in bilirubin, serum creatinine, urea, and C-reactive protein levels were observed at 28 days. Survival rates were highest with single organ failure (35.94%) and decreased with multiple organ failures. The overall survival rate was 51.56%. Predictive validity for mortality was assessed using the Area Under the Curve (AUC), with Child-Turcotte-Pugh (CTP) at 0.679, Model for End-Stage Liver Disease (MELD) at 0.819, and Chronic Liver Failure-Sequential Organ Failure Assessment (CLIF-SOFA) at 0.771.

Conclusion

This study concludes that in acute-on-chronic liver failure, factors like age, gender, and disease etiology do not significantly predict 28-day mortality. Key mortality indicators include clinical parameters such as lower Glasgow Coma Scale scores, hepatic encephalopathy Grade IV, and laboratory findings like elevated bilirubin and serum creatinine. The MELD score is the most compelling prognostic tool.

## Introduction

Acute-on-chronic liver failure (ACLF) is a distinct clinical syndrome emerging against the backdrop of chronic liver disease (CLD), marked by abrupt hepatic insults leading to significant organ dysfunction and increased mortality [1,2]. Globally, the prevalence of ACLF is about 35% among those with decompensated cirrhosis, with the highest incidence in South Asia. Mortality rates within 90 days reach 58%, rising to 73% in South America [3]. Alcohol-related liver disease is the primary etiology, while infections are common precipitants, and renal impairment is the most frequent organ dysfunction [4].

The Asian Pacific Association for the Study of the Liver (APASL) defines ACLF as an acute liver dysfunction identified by the onset of jaundice (with bilirubin levels exceeding 5 mg/dL) and coagulopathy (an International Normalized Ratio (INR) exceeding 1.5), which frequently leads to additional complications like ascites and encephalopathy in patients already suffering from chronic liver diseases. In contrast, the European Association for the Study of the Liver - Chronic Liver Failure (EASL-CLIF) Consortium describes ACLF as a sudden worsening of cirrhosis, marked by the failure of one or more organs and associated with a mortality rate of at least 15% within the initial 28-day period [5,6].

Chronic liver diseases are predominantly caused by chronic hepatitis B and C virus infections, particularly in regions such as Pakistan [7]. ACLF acute exacerbations may result from hepatic events such as acute viral hepatitis or non-hepatic sources like respiratory or urinary infections, and non-infectious factors including adverse drug reactions and surgical complications. In 40% of ACLF cases, the causes remain undetermined [1,8,9].

Predicting outcomes in ACLF requires assessing hepatic severity and multiorgan dysfunction. While traditional liver-centric scores like Child-Turcotte-Pugh (CTP) and the Model for End-Stage Liver Disease (MELD) offer some insight, they are less comprehensive than multi-organ failure assessments such as Acute Physiology and Chronic Health Evaluation II (APACHE II) and Sequential Organ Failure Assessment (SOFA), which provide superior prognostic accuracy [10,11]. The APASL's APASL-ACLF Research Consortium (AARC) AARC-ACLF score, introduced in 2017, has enhanced mortality predictions, especially in Asian populations, highlighting the need for a multifaceted evaluation framework to understand ACLF's complex clinical picture [12].

Acute-on-chronic liver failure constitutes a significant etiology for intensive care unit admissions and is associated with high mortality in chronic liver disease. The definitive therapy, liver transplantation, is often limited by economic, logistic, and expertise barriers. Consequently, this necessitates enhanced surveillance and intensive therapeutic strategies. The current investigation was designed to systematically characterize the etiological and clinical spectrum of ACLF and assess outcomes. This study identified the precipitating factors and the application of various predictive markers for risk assessment evidence-based clinical decision-making. This study aimed to target the existing lacuna in ACLF research, offering insights that could refine patient management plans.

## Materials and methods

The study sample comprised 64 patients who fulfilled the diagnostic criteria for acute-on-chronic liver failure and were admitted to the Gastroenterology department at Lahore General Hospital between November 2022 and August 2023. Ethical approval for the study was obtained from the Institutional Ethical Review Committee of the Post Graduate Medical Institute/Ameer-Ud-Din Medical College, Lahore General Hospital (AMC/PGMI/LGH/316/31/10/2022) and informed consent was acquired from all participants or their legal guardians if the patients were unable to provide consent. The sample size was determined based on a predicted 40% mortality rate in ACLF, with the aim of achieving statistical significance with an 80% power and 5% type I margin of error. A non-probability consecutive sampling technique was used for participant selection [8].

Patients aged 18 to 70 years having jaundice, coagulopathy (INR >1.5), ascites, and/or encephalopathy within four weeks on a background of chronic liver disease, as defined by the European Association for the Study of the Liver - Chronic Liver Failure (EASL-CLIF) definition, were included in the study. Diagnosis of chronic liver disease was confirmed by evidence of heterogenous liver parenchyma or surface irregularity on ultrasonography, splenomegaly, or a hepatic venous pressure gradient exceeding 10 mmHg indicative of portal hypertension, esophageal varices identified during endoscopy, or a FibroScan assessment revealing fibrosis with values greater than 7.0 kPa, corresponding to significant fibrosis (≥F2). Participants with previously known organ failures such as end-stage renal disease or congestive heart failure, non-hepatic organ dysfunctions, portal vein thrombosis, and malignancies, including hepatocellular carcinoma were excluded. Additionally, patients with a history of drug-induced liver injury without pre-existing CLD, HIV infection, and chronic decompensation evident by multiple hospitalizations during the last six months were not included in the study.

During data collection, an in-depth demographic analysis was followed by a careful review of patients' clinical histories, highlighting comorbidities and the chronicity of liver disease. Pharmacological histories and hepatotoxic risk factors were given attention to discern precipitants of acute deterioration in chronic hepatic conditions. Clinical evaluations noted vital signs, jaundice, and neurological status, using the Glasgow Coma Scale, and observed for hepatic or splenic enlargement, ascites, and other signs of long-standing liver disease. Initial lab tests encompassed complete blood counts, renal and hepatic panels, coagulation profiles, and microbial cultures from blood and urine samples. Serological testing for hepatitis viruses A through E, chest X-rays, and abdominal ultrasonography were conducted. Where patient histories suggested metabolic or autoimmune etiologies, specific assays for Wilson's disease, hemochromatosis, and autoimmune liver profiles were performed, aiding in chronic liver disease diagnosis and acute-on-chronic liver failure etiology identification.

Acute-on-chronic liver failure (ACLF) was classified according to the EASL-CLIF consortium criteria as follows: ACLF grade 1 is characterized by single-organ dysfunction. The presence of two organ failures is defined as ACLF grade 2, and three or more organ failures are identified as ACLF grade 3. All the patients were classified at the enrollment. Organ failure was delineated utilizing the CLIF-SOFA criteria, which demarcated renal impairment at a threshold of serum creatinine ≥2 mg/dL, hepatic insufficiency at serum bilirubin levels ≥12 mg/dL, neurological compromise at hepatic encephalopathy grades 3 or 4 following West Haven conventions, circulatory collapse necessitating vasoactive support, coagulopathy with an INR ≥2.5, and pulmonary dysfunction characterized by either a peripheral oxygen saturation (SpO2)/fraction of inspired oxygen (FiO2) ratio ≤200 or the initiation of artificial ventilation. Hepatic encephalopathy severity was as follows: Grade 1, characterized by a mild alteration in consciousness with anxiety or euphoria and attention deficits; Grade 2, involving moderate consciousness impairment with lethargy or apathy and disorientation; Grade 3, severe consciousness disturbance with confusion and semi stupor but responsive to stimuli; and Grade 4, a critical state of unresponsiveness or coma.

Sepsis is characterized by one or more of the following clinical criteria: hyperthermia (>38°C) or hypothermia (<35°C), tachycardia (>90 bpm), tachypnea (>20 breaths/min), and leukocytosis (>12,000 cells/mm³), leukopenia (<4,000 cells/mm³). CTP, MELD, and CLIF-SOFA scores were determined for each subject in the study using MedCalc software (MedCalc Software Ltd., Ostend, Belgium). Patient management adhered to a multi-faceted protocol, providing escalated care from conservative to intensive as indicated. Sepsis was initially addressed with broad-spectrum antibiotics, refined by microbiological profiling. Spontaneous bacterial peritonitis was treated according to the established guidelines. Therapeutic paracentesis was conducted when necessary. Renal and respiratory support were instituted based on clinical indication. Encephalopathy and hypotension were managed with anti-encephalopathy measures and inotropes, respectively. Esophageal varices were treated with Endoscopic Variceal Ligation (EVL) within 24 hours, supplemented by protocol-driven gastrointestinal bleed management. Patients were counseled regarding the option of liver transplantation. Patients were systematically observed over four weeks to record mortality within 28 days post-diagnosis of acute-on-chronic liver failure (ACLF). Documentation was meticulously carried out using a structured data collection proforma.

Statistical evaluation was performed using SPSS software, version 26.0 (IBM Corp., Armonk, USA). Continuous data, including patient age, chronic liver disease duration, mean arterial pressure, and lab test results were expressed as median and interquartile ranges (IQR). Frequency counts and percentages described categorical data such as sex, existing comorbid conditions, and the origin of liver disease. Odds ratios with 95% CI were calculated for binary and categorical data to determine the magnitude and direction of their association with patient outcomes. Chi-Square or Fisher's Exact Test was applied to explore the relationship between categorical factors and patient survival rates, contingent on the data's distribution. The Mann-Whitney U test was used to compare continuous variables between patient groups with different survival outcomes. The prognostic performance of various scoring systems (CTP, MELD, and CLIF-SOFA) and baseline lab results in predicting 28-day mortality were evaluated via Receiver Operating Characteristic (ROC) curves. The AUC measured the overall predictive accuracy of each scoring system in differentiating survivors from non-survivors. Sensitivity and specificity were identified at the most discriminating threshold values. A p-value of less than 0.05 was considered indicative of statistical significance.

## Results

Table 1 reveals that the median age and duration of chronic liver disease did not differ significantly between survivors and non-survivors of ACLF, and mortality was not significantly influenced by gender or comorbidities. Non-survivors presented with a lower median arterial pressure; however, the difference was insignificant. Significantly higher mortality correlated with lower Glasgow Coma Scale scores, Grade IV hepatic encephalopathy, and upper gastrointestinal bleeding. 

**Table 1:** Comparison of baseline demographic, etiological, and clinical details between Survivors and Non-Survivors COPD: chronic obstructive pulmonary disease; CLD: chronic liver disease; NASH: non-alcoholic steatohepatitis; HBV: hepatitis B virus; HCV: hepatitis C virus; HAV: hepatitis A virus; HEV: hepatitis E virus; GSC: Glasgow Coma Scale; CTP: Child-Turcotte-Pugh

Baseline Characteristics	Survivor	Non-Survivor	Odds Ratio (95% CI)	p-value
Age Median, years (IQR-Range)	54.00 (22-43)	56.00 (13-50)	'-'	0.35
Duration of Chronic Liver Disease (years) Median (IQR-Range)	5.00 (2-6)	5.00 (2-4)	'-'	0.913
Gender – Male	17 (26.6%)	22 (34.4%)	0.435 (0.155-1.221)	0.111
Gender – Female	16 (25%)	9 (14%)		
Comorbidities				
Diabetes	4 (6.25%)	6 (9.38%)	10 (15.62%)	0.542
Hypertension	3 (4.69%)	7 (10.94%)	10 (15.62%)	
Diabetes plus Hypertension	7 (10.94%)	3 (4.69%)	10 (15.62%)	
Tuberculosis	3 (4.69%)	2 (3.12%)	5 (7.81%)	
Asthma/COPD	5 (7.81%)	4 (6.25%)	9 (14.06%)	
None	11 (17.19%)	9 (14.06%)	20 (31.25%)	
Etiology of CLD				
HBV	6 (9.38%)	7 (10.94%)	13 (20.31%)	0.936
HCV	17 (26.56%)	17 (26.56%)	34 (53.12%)	
Autoimmune	1 (1.56%)	1 (1.56%)	2 (3.12%)	
NASH	2 (3.12%)	2 (3.12%)	4 (6.25%)	
Cryptogenic	1 (1.56%)	1 (1.56%)	2 (3.12%)	
HBV plus HCV	4 (6.25%)	1 (1.56%)	5 (7.81%)	
Alcoholic Liver Disease	2 (3.12%)	2 (3.12%)	4 (6.25%)	
Causes of acute on chronic liver failure				
Acute Viral Hepatitis (HAV)	4 (6.25%)	7 (10.94%)	11 (17.19%)	0.501
HDV Superinfection	2 (3.12%)	0 (0.00%)	2 (3.12%)	
Surgery	1 (1.56%)	2 (3.12%)	3 (4.69%)	
Acute Viral Hepatitis (HEV)	2 (3.12%)	3 (4.69%)	5 (7.81%)	
Malignancy	3 (4.69%)	3 (4.69%)	6 (9.38%)	
Spontaneous Bacterial Peritonitis	11 (17.19%)	7 (10.94%)	18 (28.12%)	
Drugs	3 (4.69%)	4 (6.25%)	7 (10.94%)	
Dengue	1 (1.56%)	3 (4.69%)	4 (6.25%)	
Urinary Tract Infection	1 (1.56%)	1 (1.56%)	2 (3.12%)	
Respiratory Tract Infection	1 (1.56%)	1 (1.56%)	2 (3.12%)	
Unknown	4 (6.25%)	0 (0.00%)	4 (6.25%	
Clinical presentation				
Mean Arterial Pressure Median mmHg (IQR-Range)	71.00 (14-41)	63.00 (17-50)		0.092
Temperature (<95 °F)	8 (12.50%)	10 (15.62%)	18 (28.12%)	0.156
Temperature (95 °F to 99.5 °F)	11 (17.19%)	4 (6.25%)	15 (23.44%)	
Temperature (>99.5 °F)	14 (21.88%)	17 (26.56%)	31 (48.44%)	
GCS				
GCS 1-3	1 (1.56%)	9 (14.06%)	10 (15.62%)	0.022
GCS 4-8	7 (10.94%)	8 (12.50%)	15 (23.44%)	
GCS 9-12	14 (21.88%)	7 (10.94%)	21 (32.81%)	
GCS 13-15	11 (17.19%)	7 (10.94%)	18 (28.12%)	
Upper GI Bleed	7 (10.94%)	14 (21.88%)	0.327 (0.109-0.976)	0.041
Splenomegaly	17 (26.56%)	20 (31.25%)	0.584 (0.214-1.594)	0.293
Hepatomegaly	18 (28.12%)	16 (25.00%)	1.125 (0.421-3.006)	0.814
Ascites - Mild	7 (10.94%)	5 (7.81%)	12 (18.75%)	0.819
Ascites - Moderate	14 (21.88%)	12 (18.75%)	26 (40.62%)	
Ascites - Gross	9 (14.06%)	9 (14.06%)	18 (28.12%)	
Ascites - None	3 (4.69%)	5 (7.81%)	8 (12.50%)	
Hepatic Encephalopathy Grade I	4 (6.25%)	4 (6.25%)	8 (12.50%)	0.035
Hepatic Encephalopathy Grade II	14 (21.88%)	7 (10.94%)	21 (32.81%)	
Hepatic Encephalopathy Grade III	7 (10.94%)	8 (12.50%)	15 (23.44%)	
Hepatic Encephalopathy Grade IV	1 (1.56%)	9 (14.06%)	10 (15.62%)	
No Hepatic Encephalopathy	7 (10.94%)	3 (4.69%)	10 (15.62%)	
CTP Class - A	13 (20.31%)	7 (10.94%)	20 (31.25%)	0.013
CTP Class - B	16 (25.00%)	10 (15.62%)	26 (40.62%)	
CTP Class - C	4 (6.25%)	14 (21.88%)	18 (28.12%)	

Table 2 compares laboratory values between ACLF survivors and non-survivors at 28 days, revealing significant disparities in bilirubin, serum creatinine, urea, and C-reactive protein levels, and prognostic scores such as CTP, MELD-Na, and CLIF-SOFA, suggesting their prognostic relevance. Other parameters showed no significant differences.

**Table 2:** Comparison of Baseline Laboratory Parameters and Scores among Survivors and Non-survivors AST: aspartate aminotransferase; ALT: alanine aminotransferase; INR: International Normalized Ratio; CTP: Child-Turcotte-Pugh; MELD: Model for End-Stage Liver Disease; CLIF-SOFA: Chronic Liver Failure-Sequential Organ Failure Assessment

Laboratory Parameters and Scores	Survivor Median, IQR (Range)	Non-Survivor Median, IQR (Range)	p-value
Hemoglobin	9, 5 (7 - 14)	9, 5 (7 - 14)	0.788
WBC	12, 9 (3 - 18)	12, 15 (3 - 27)	0.532
Platelet	100, 63 (35 - 210)	105, 90 (26 - 215)	0.793
AST	251, 219 (58 - 8915)	254, 2632 (99 - 8600)	0.432
ALT	244, 175 (76 - 8665)	240, 2260 (110 - 5993)	0.32
Bilirubin	1, 3 (0 - 18)	7, 13 (1 - 18)	0.002
Albumin	3, 1 (2 - 4)	3, 2 (2 - 4)	0.273
Prothrombin Time	14, 5 (10 - 19)	16, 3 (10 - 18)	0.222
INR	2, 1 (1 - 3)	2, 1 (1 - 3)	0.106
Serum Creatinine	2, 2 (1 - 9)	3, 2 (1 - 9)	0.006
Urea	88, 48 (43 - 235)	160, 100 (47 - 278)	0.000
Serum Sodium	134, 8 (123 - 152)	132, 10 (119 - 150)	0.228
C-Reactive Protein	35, 38 (15 - 116)	65, 35 (23 - 120)	0.001
CTP Score	7, 3 (5 - 11)	9, 4 (5 - 14)	0.013
MELD Score	26, 8 (13 - 38)	32, 7 (21 - 43)	0.000
CLIF-SOFA Score	45, 9 (28 - 55)	53, 14 (38 - 66)	0.000

Table 3 detailed the relationship between organ dysfunction, measured by the CLIF-C SOFA score, and patient survival outcomes. 

**Table 3:** Impact of Organ Involvement on Survival Outcomes in CLIF-C SOFA Score Assessment PaO2: peripheral oxygen saturation; FiO2: fraction of inspired oxygen; MAP: mean arterial pressure; INR: international normalized ratio

Organ Involvement	Survivor	Non-Survivor	p-value
Renal Failure - Creatinine < 2	13	6	0.006
Renal Failure - Creatinine 2.0 to < 3.5	19	15	
Renal Failure - Creatinine >= 3.5	1	10	
Respiratory Failure - PaO2/FiO2 > 300	19	14	0.034
Respiratory Failure - PaO2/FiO2 200-300	11	6	
Respiratory Failure - PaO2/FiO2 <= 200 or intubated	3	11	
Circulatory Failure - MAP >= 70	18	6	0.007
Circulatory Failure - MAP < 70	9	10	
Circulatory Failure - Any MAP with vasopressor	6	15	
Hepatic Failure - Bilirubin < 6	27	15	0.019
Hepatic Failure - Bilirubin 6 to < 12	3	7	
Hepatic Failure - Bilirubin >= 12	3	9	
Coagulation Failure - INR <2	19	11	0.081
Coagulation Failure - INR 2-2.5	9	17	
Coagulation Failure - INR >=2.5	5	3	
Cerebral Failure - Present	7	20	<0.001
Cerebral Failure - Absent	26	11	

**Figure 1:**
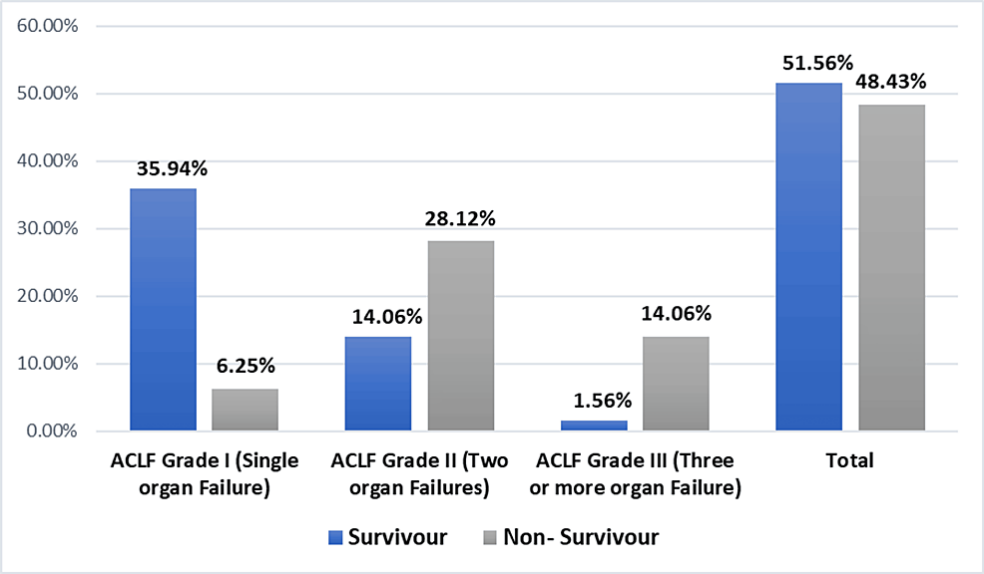
Survival outcomes at 28 days by acute-on-chronic liver failure (ACLF) grade

Figure 1 demonstrates the correlation between organ involvement and 28-day survival versus mortality, with a significant p-value (<0.001).

**Figure 2:**
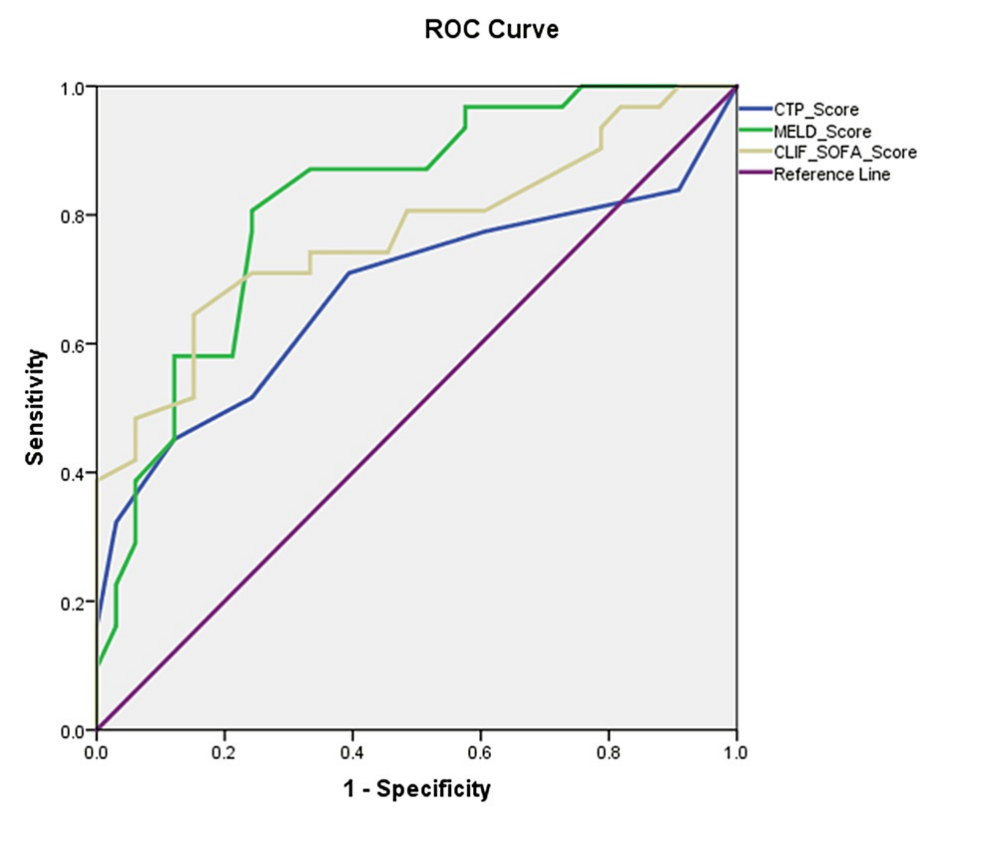
Comparative analysis of CTP, MELD, and CLIF-SOFA scoring systems in predicting mortality: an ROC curve assessment CTP: Child-Turcotte-Pugh; MELD: Model for End-Stage Liver Disease; CLIF-SOFA: Chronic Liver Failure-Sequential Organ Failure Assessment; ROC: receiver operating characteristic

In the present study, ROC curve analysis was employed to ascertain the discriminatory capacity of the CTP Score, MELD Score, and CLIF-SOFA Score in predicting mortality among patients with acute-on-chronic liver failure. The AUC for the CTP Score was 0.679 (95% CI, 0.542-0.817; p = 0.014), for the MELD Score was 0.819 (95% CI, 0.715-0.922; p < 0.001), and for the CLIF-SOFA Score was 0.771 (95% CI, 0.653-0.889; p < 0.001), indicating that the MELD Score had the highest discriminative ability among the three scores evaluated (Figure 2). Threshold analysis yielded a CTP Score cutoff of 9.50, manifesting a sensitivity of 45.2% and a specificity of 87.9%. Conversely, a MELD Score threshold of 21.50 was marked by a high sensitivity of 96.8%, although specificity was relatively diminished at 27.3%. The CLIF-SOFA Score, at a cutoff of 43.50, balanced sensitivity and specificity at 83.9% and 33.3%, respectively. Notable findings were observed in the ROC analysis evaluating baseline laboratory parameters for predicting 28-day mortality. Bilirubin, urea, and C-reactive protein were significant discriminative markers, with respective AUC values of 0.730, 0.753, and 0.742, all indicating diagnostic potential. Serum creatinine also emerged as a significant indicator (AUC=0.701). The International Normalized Ratio (INR) showed moderate discrimination, while alanine transaminase (ALT) and aspartate transaminase (AST) were less effective.

## Discussion

In the present analysis, the age distribution within survivor and non-survivor cohorts of ACLF did not show a significant difference, with medians at 54 and 56 years, respectively (p=0.35). This finding is in accordance with Reddy et al. (2021) [12], where ages for survivors and non-survivors were 35.97 ± 8 years and 39.76 ± 8.7 years (p=0.15), and Saxena et al. (2020) [13], who reported ages of 36 ± 7 years for survivors and 40 ± 6 years for non-survivors with a non-significant p-value (>0.05).

Shin et al. [14], in their study, recorded a median age of 54.8 years among participants, noting it did not markedly influence mortality rates (Hazard Ratio (HR) 1.00, 95% Confidence Interval (CI): 0.98-1.03, p=0.828) [14]. Saad et al. (2022) [15] reported a higher mean age of 62 ± 7.2 years. Kulkarni et al. (2018) [16] observed median ages of 42 years for those who survived and 47 years for those who did not (p=0.667) [16,17]. Tasneem et al. (2017) [7] reported that individuals over 40 years exhibited a higher risk of mortality (p=0.036, Odds Ratio [OR] = 0.325). These findings collectively indicate that age alone may not be a dependable predictor of mortality in ACLF. Thus, the study concludes that age is not a significant independent prognostic indicator for mortality in patients with ACLF.

In the current research, the distribution of participants was 61% male to 39% female, which did not present a statistically significant variance in survival outcomes (p=0.111, Odds Ratio [OR] 0.435 [0.15-1.22]). This aligns with gender distributions noted in the existing literature. Saad et al. [15] identified a sample distribution of 67% male to 33% female, whereas Behera et al. (2021) [17] found a male-to-female ratio 2.5:1. Reddy et al. (2021) [12] reported a ratio of 3.7:1 among survivors and 4.2:1 among non-survivors (p=0.820), and El Sayed et al. (2021) [8] likewise observed no significant gender-based differences in survival (p=0.721). Shin et al. (2020) [14] determined that male gender did not substantially affect mortality (HR 0.65 [0.27-1.56], p=0.335). In contrast, Saxena et al. (2010) [13] found a significant difference, with 19% males in survivors versus 1% females and 29% males in non-survivors versus 1% females (p=0.02). Kulkarni et al. [16] reported a non-significant gender difference (p=0.720), and Tasneem et al. (2017) [7] echoed the absence of statistical significance (p=0.528, OR=1.418 [0.479-4.202]). The cumulative evidence suggests that while male prevalence is higher in ACLF, gender does not significantly influence mortality, which aligns with the current study's findings, reinforcing the notion that gender alone is not a determinant of survival in ACLF.

In the current study, the mortality rate was reported at 48.4%, aligning closely with the range of mortality rates noted in various studies on acute-on-chronic liver failure (ACLF). Reddy et al. (2021) [12] reported a rate of 43.33%, Behera et al. (2021) [17] 46.6%, and Saad et al. (2022) [15] reported a mortality rate of 30% [13,16,18]. Similarly, Kulkarni et al. (2018) [16] reported a death rate of 43.75%, while Garg et al. (2012) [18] and Mikolasevic et al. (2015) [19] both reported higher mortality rates of 50%. Tasneem et al. (2017) [7] recorded a slightly low death rate of 39.3% compared to this and other previous literature. Collectively, these findings underscore the significant mortality burden associated with ACLF.

In this research, Hepatitis C Virus (HCV) emerged as the predominant cause of Chronic Liver Disease (CLD), accounting for 53.12% of cases, followed by Hepatitis B Virus (HBV) at 20.3%. Autoimmune and cryptogenic sources were identified as the least frequent, constituting 3.12% of the etiological factors. Nonetheless, these causative factors did not significantly influence the survival rates (p > 0.05). In contrast, Mikolasevic et al. in 2015 [19] identified alcohol as the leading cause of CLD in 77.7% of their study population, while Garg et al. in 2012 [18] and Tasneem et al. in 2017 [7] reported HBV as the most prevalent etiology, with incidences of 37% and 25% respectively. Similarly, Saxena et al. (2020) [13] and Behera et al. (2021) [17] also documented that alcohol was the most common etiology of CLD, followed by HBV and HCV. Saad et al. (2022) [15] reported a high prevalence of HCV at 78%, similar to the current study. The differences in etiological factors across studies underline the geographical and demographic variability in CLD causation. Despite the differences, etiology was not a significant factor affecting mortality in this study.

In the current study, spontaneous bacterial peritonitis (SBP) was the predominant precipitating factor for ACLF at 28.12%, with acute viral hepatitis A and drug-induced liver injury following at 17.19% and 10.94%, respectively. Yet, these factors did not significantly influence outcomes. This contrasts with Tasneem et al. (2017) [7], who highlighted acute viral hepatitis, primarily hepatitis E virus (HEV), as the leading cause at 33.3%, and Saad et al. (2022) [15], who reported drugs as the primary etiology at 33%. El Sayed et al. (2021) [8] and Behera et al. (2021) [17] identified SBP and alcoholic hepatitis as major factors, with the latter noting significant outcome differences in relation to alcoholic hepatitis (p=0.017) and upper gastrointestinal (GI) bleeding (p=0.002). Saxena et al. (2020) [13] reported alcohol as the leading cause at 50%. The disparities in ACLF precipitating factors are primarily attributable to regional differences; in India and Western countries, alcohol is a significant factor, whereas in Pakistan, its consumption is less prevalent due to religious practices.

In this study, jaundice was universal, with ascites being the second most common manifestation at 88%. Mean arterial pressure (MAP) differed between non-survivors (63 mmHg) and survivors (71 mmHg), albeit non-significantly (p=0.092). Neither temperature variation nor the presence of ascites impacted the 28-day mortality outcome (p > 0.05). Additionally, hepatomegaly and splenomegaly did not significantly influence survival, with respective ORs of 1.125 (p=0.814) and 0.584 (p=0.293). However, a Glasgow Coma Scale (GCS) score of 3 or lower was associated with higher mortality (14% vs 1.5% in survivors, p=0.022). Hepatic encephalopathy (HE) was present in 95% of patients, with higher survival observed in those with no or mild HE (grades 1-2) compared to those with severe HE (grade 4).

Other studies have identified varying prevalences of ascites and HE, with Saad et al. (2022) [15] reporting 59% and 18.5% for ascites and HE, respectively, while Behera et al. (2021) [17] found ascites in 88% and HE in 60% of their patients. Shin et al. (2020) [14] indicated ascites as a risk factor for mortality (HR 2.99, p=0.013), though HE was less common at 13% [15]. Saxena et al. (2020) [13] observed ascites in 88% and jaundice in 100% of patients, with severe HE more prevalent in the death group (77% vs 22%, p=0.01) [14]. Similarly, Tasneem et al. (2017) [7] linked advanced HE to increased mortality (p=0.001, OR 11.82), a finding echoed by Reddy et al. (2021) [12], Kulkarni et al. (2018) [16], and other studies that recognized high-grade HE as an independent predictor of mortality. Collectively, while some clinical features such as MAP and ascites did not display a significant association with outcomes, severe HE consistently emerged as a critical determinant of mortality in ACLF, underscoring its potential as a prognostic indicator.

In the current study, notable differences were observed in urea, MELD Score, and CLIF-SOFA Score, all showing strong statistical significance for mortality (p = 0.000). Bilirubin and C-Reactive Protein also significantly differed (p = 0.002 and p = 0.001, respectively). Serum Creatinine and CTP Score showed moderate significance (p = 0.006 and p = 0.013). Conversely, INR, Serum Sodium, Prothrombin Time, Albumin, AST, WBC, Platelet Count, Hemoglobin, and ALT were insignificant (p > 0.05). Comparatively, Behera et al. (2021) [17] reported significant differences in Total Leucocyte Count, Total Bilirubin, Serum Albumin, INR, Serum Creatinine, and Serum Sodium (p = 0.00001), and in CTP Class and MELD Score (p = 0.0001). Reddy et al. (2021) [12] found the MELD Score, SOFA Score, Serum Creatinine, Total Bilirubin, and Albumin to be significant predictors of mortality. Shin et al. (2020) [14] identified WBC, Albumin, total bilirubin, prothrombin time (PT), sodium, creatinine, MELD score, CTP score, and CLIF-SOFA score as significant (p < 0.01). Saxena et al. (2020) [13] reported significant differences in hemoglobin (HB), TLC, Urea, Creatinine, INR, total bilirubin, and AST. Tasneem et al. (2017) [7] highlighted CTP Score (p = 0.010, OR = 4.286), MELD Score (p = 0.001, OR = 6.222), Albumin (p = 0.116, OR = 2.708), and INR (p = 0.075, OR = 2.585). Kumar et al. (2016) [20] noted significant differences in Prothrombin Time, INR, Creatinine, AST, s/bilirubin, Sodium, and ALT. Kulkarni et al. (2018) [16] found Sodium, Potassium, Creatinine, Hemoglobin, and INR to be significant. The current study and existing literature demonstrated that the MELD Score and Serum Creatinine consistently emerged as significant predictors of mortality in ACLF (p-values consistently < 0.01 across studies). Similarly, there was a significant difference in Bilirubin and INR levels between survivors and non-survivors across multiple studies. In contrast, parameters like AST, ALT, and hemoglobin showed limited utility as independent prognostic markers of mortality among ACLF patients.

This study presents a mortality rate of 48.4%, with escalating rates from ACLF Grade I (0%) to Grade III (14.06%). Comparable trends are evident in the previous literature, with higher ACLF grades correlating with increased mortality [16-18]. Kumar et al. (2016) [20] reported mortality rates of 90% with three or more organ failures. Similarly, Reddy et al. (2021) [12] also observed significantly greater mortality in Type C ACLF. According to these studies, higher ACLF grades and multiorgan involvement have prognostic significance.

The current study's ROC curve analysis reported the MELD Score as the strongest predictor of mortality with an AUC of 0.819, higher than the CTP Score (AUC: 0.679) and the CLIF-SOFA Score (AUC: 0.771). Contrarily, Kulkarni et al. (2018) [16] reported findings with the MELD Score showing an AUC of 0.783 and a significant p-value (<0.0001), alongside the CLIF-SOFA and CTP Scores with AUCs of 0.947 and 0.795 respectively, both with p-values <0.0001, showing MELD score underperformed compared to others. Garg et al. (2012) [18] presented an AUC of 0.667 for the CTP score, contrasting with higher AUCs for MELD, SOFA, and APACHE-II scores, all exceeding 0.8. Saxena et al. (2020) [13] highlighted the CLIF-SOFA and MELD scores as the most reliable for predicting the 28-day outcome of ACLF, while Mikolasevic et al. (2015) [19] reported the APACHE II score with an AUC of 0.878 as the best predictor of short-term mortality. Kumar et al. (2016) [20] demonstrated that the MELD score had a slight advantage over SOFA, with an AUC of 0.727 compared to SOFA's AUC of 0.636. These results highlighted the variability in prognostic accuracy among different scoring systems for ACLF, with the MELD Score being consistent as a key predictor of mortality. These findings support the role of the MELD Score as a reliable predictive tool for mortality among patients with ACLF, considering its demonstrated efficacy across diverse patient populations and clinical settings.

The limitations of this study include its confinement to a single center, the relatively modest number of participants, and the absence of longitudinal analysis. Nevertheless, its robustness is anchored in the comparative assessment of recognized prognostic indicators, where the score in question was deemed the most accurate in forecasting mortality, aligning with prior studies. The diversity in disease etiology also accentuates the impact of geographic variables on health outcomes. To enhance the generalizability of these findings, future investigations should adopt multicentric methodologies that integrate a broader spectrum of patient populations.

## Conclusions

This study concludes that demographic variables such as age, gender, and the underlying etiology of liver disease are not predictive of 28-day in-hospital mortality in acute-on-chronic liver failure. Significant clinical determinants of mortality include Glasgow Coma Scale scores and advanced hepatic encephalopathy, alongside laboratory markers like bilirubin and serum creatinine levels. The prognostic accuracy of the Model for End-Stage Liver Disease Score surpasses other scoring systems, establishing it as a superior predictive tool for assessing mortality risk within this patient population. These insights underscore the necessity of focusing on clinical assessments and specific laboratory parameters to predict short-term outcomes in ACLF.
